# Power Doppler ultrasonographic assessment of the joint-draining lymph node complex in rheumatoid arthritis: a prospective, proof-of-concept study on treatment with tumor necrosis factor inhibitors

**DOI:** 10.1186/s13075-016-1142-7

**Published:** 2016-10-22

**Authors:** Antonio Manzo, Francesca Benaglio, Barbara Vitolo, Chandra Bortolotto, Francesca Zibera, Monica Todoerti, Claudia Alpini, Serena Bugatti, Roberto Caporali, Fabrizio Calliada, Carlomaurizio Montecucco

**Affiliations:** 1 Rheumatology and Translational Immunology Research Laboratories (LaRIT) and Biologic Therapy Unit, Division of Rheumatology, IRCCS Policlinico San Matteo Foundation/University of Pavia, P.le Golgi 19, 27100 Pavia, Italy; 2 Division of Radiology, IRCCS Policlinico San Matteo Foundation/University of Pavia, Pavia, Italy; 3 Laboratory of Biochemical-Clinical Analyses, IRCCS Policlinico San Matteo Foundation, Pavia, Italy

**Keywords:** Lymph node, Ultrasonography, Rheumatoid arthritis

## Abstract

**Background:**

Emerging research on the mechanisms of disease chronicity in experimental arthritis has included a new focus on the draining lymph node (LN). Here, we combined clinical-serological analyses and power Doppler ultrasound (PDUS) imaging to delineate noninvasively the reciprocal relationship in vivo between the joint and the draining LN in patients with rheumatoid arthritis (RA).

**Methods:**

Forty consecutive patients refractory to conventional synthetic disease-modifying anti-rheumatic drugs were examined through parallel PDUS of the hand–wrist joints and axillary LNs and compared with 20 healthy subjects. A semiquantitative score for LN gray-scale (GS) parameters (nodal hypertrophy and cortical structure) and LN PD signal was developed. A 6-month follow-up study with serial sonographic assessments was then performed on initiation of tumor necrosis factor (TNF) inhibitors.

**Results:**

PDUS analysis of RA axillary LNs revealed the existence of marked inter-individual heterogeneity and of quantitative differences compared with healthy individuals in both GS and PD characteristics. RA LN changes were plastic, responsive to anti-TNF treatment, and displayed a degree of concordance with synovitis activity in peripheral joints. However, low LN PD signal at baseline despite active arthritis was strongly associated with a poor clinical response to TNF blockade.

**Conclusions:**

PDUS analysis of the draining LN in RA allows capture of measurable inter-individual differences and dynamic changes linked to the underlying pathologic process. LN and joint sonographic assessments are nonredundant approaches that may provide independent perspectives on peripheral disease and its evolution over time.

**Electronic supplementary material:**

The online version of this article (doi:10.1186/s13075-016-1142-7) contains supplementary material, which is available to authorized users.

## Background

The juxta-articular lymphoid system (JLS) is a complex of immunocompetent structures composed of the afferent lymphatic network and the draining lymph node (LN) chains [1]. These are fixed environments functionally connected to the periphery, acting as complementary checkpoints through progressive steps of the inflammatory cascade, including the egress of cells and fluids from the periphery [2], lymphocyte polarization and memory imprinting [3], and peripheral tolerization [4, 5]. In keeping with these concepts, the JLS has been shown to play key roles in experimental arthritis, both in the development of arthritogenic autoimmunity [6] and in the remote control of peripheral inflammation through compensatory drainage [7–9].

Circumstantial evidence supporting the participation of the draining LN in rheumatoid arthritis (RA) derives from independent studies performed over the last decades. Lymphadenopathy has been recognized as a possible extra-articular manifestation of the disease [10]. ^18^F-FDG PET hypercaptation in axillary LN is detectable in patients with active pathology [11]. Accordingly, prenodal lymph from RA joints is characterized by increased flow rate and cytokine concentration compared with controls [12]. Despite these data, the relationship between chronic synovitis and JLS involvement, including its clinical significance, remains almost completely unexplored.

One of the main challenges in this direction is the limitation in assessing the joint-draining LN complex in vivo through multisite and serial analyses. Power Doppler ultrasonography (PDUS) is a cheap, rapid, noninvasive, and sensitive imaging technique extensively used to visualize signs of joint inflammatory activity [13]. These signs include tissue hypertrophy and altered microperfusion assessed through codification of the power spectral density of the Doppler signal [14]. Of note, tissue swelling and vascular flow enhancement are not restricted to inflamed peripheral tissues, but are similarly induced during LN immune-inflammatory challenge. Nodal response to lymph-borne stimuli actually involves a sequence of plastic events characterized by remodeling of the feed arteriole [15], increased lymphocyte recruitment [15], expansion of the vascular-stromal compartment [16, 17], and decreased cell exit (“shut-down”) [18], ultimately leading to increased blood flow, enlargement of the lymphocyte-rich cortex, and LN hypertrophy [15, 19–22]. Supporting the value of PDUS to visualize these processes, the sonographic assessment of nodal dimensions, internal structure, and perfusion is an established component of cancer diagnostic work-up, being exploited to screen signs of metastasis or inflammatory reactivity [23].

We have recently obtained preliminary evidence that axillary LN PDUS can allow the detection of qualitative modifications also in patients with RA [24]. To what extent the analysis of the draining LNs can be applied to delineate inter-individual differences or dynamic changes in the course of the disease, and whether it provides novel, relevant information, remains undetermined.

To address this question, we performed an integrated analysis of the hand, wrist, and axillary LN ultrasonographic (US) characteristics in patients with active disease, exploring prospectively two primary issues: the spectrum of structural and vascular alterations of RA axillary LNs detectable by US; and this spectrum’s relationship with the synovial inflammatory process and clinical phenotype, before and on treatment with tumor necrosis factor (TNF) inhibitors.

## Methods

### Recruitment criteria

Forty patients referred to the Biologic Therapy Unit (Rheumatology Division) of the IRCCS Policlinico San Matteo Foundation, Pavia, Italy were included (Table 1). Patients were consecutively enrolled according to the following criteria: fulfillment of the ACR 1987 classification criteria for RA [25]; no current or previous treatment with biologic therapies; inadequate response to conventional synthetic DMARDs (csDMARDs) [26]; and 28-joint Disease Activity Score (DAS28) ≥ 3.2 [27]. Oral glucocorticoids (≤7.5 mg/day of prednisone equivalents) and nonsteroidal anti-inflammatory drugs were allowed.

**Table 1:** Demographic and clinical characteristics of the patient population at baseline

	*n* = 40 patients
Age (years), mean (SD)	54.6 (14)
Females, *n* (%)	32 (80)
Disease duration (months), median (IQR)	38 (19–115)
DAS28, mean (SD)	4.87 (0.84)
SJC28, median (IQR)	4 (1.5–5.5)
TJC28, median (IQR)	8 (4–12.5)
VAS PtGA (mm), median (IQR)	65 (50–80)
HAQ-DI, median (IQR)	1.125 (0.75–1.5)
ESR (mm/1 h), median (IQR)	22 (18–36.5)
CRP (mg/dl), median (IQR)	0.9 (0.3–2.75)
12-joint GS index, median (IQR)	13 (8.5–18.5)
12-joint PD index, median (IQR)	2 (0–5.5)
IgM RF positive, *n* (%)	26 (65)
IgM RF titer (U/ml), median (IQR)^a^	85 (42.5–274)
IgG ACPA positive, *n* (%)	27 (67.5)
IgG ACPA titer (U/ml), median (IQR)^a^	66 (27.2–287.5)
Erosive disease, *n* (%)^b^	23/32 (71.9)
Current treatment with MTX, *n* (%)	36 (90)
Receiving corticosteroids, *n* (%)	31 (77.5)
Receiving NSAIDs, *n* (%)	9 (22.5)
Number of previous csDMARDs, median (range)	1 (0–3)

^a^RF or ACPA titers in RF-positive or ACPA-positive patients respectively. ACPA titers > 340 U/ml were not diluted further

^b^Hands and feet X-ray data not available in eight patients

*SD* standard deviation, *IQR* interquartile range, *DAS28* Disease Activity Score in 28 joints, *SJC28* swollen joint count in 28 joints, *TJC28* tender joint count in 28 joints, *VAS* visual analogue scale, *PtGA* patient’s global assessment, *HAQ-DI* Health Assessment Questionnaire disability index, *ESR* erythrocyte sedimentation rate, *CRP* C-reactive protein, *GS* gray scale, *PD* power Doppler, *RF* rheumatoid factor, *ACPA* anti-citrullinated peptide antibodies, *MTX* methotrexate, *NSAID* nonsteroidal anti-inflammatory drug, *csDMARD* conventional synthetic disease-modifying anti-rheumatic drug

Twenty volunteers (mean age ± standard deviation (SD): 53.2 ± 17.2 years, females: 75 %) free from chronic inflammatory arthropathies were enrolled as controls. The following exclusion criteria were applied to all participants: history of malignancies; concomitant autoimmune or infectious diseases; vaccinations and physical traumas in the preceding 4 weeks; current treatment with peripheral vasodilators; and body mass index ≥ 35 (to limit potential biases in physical examination of axillary LNs in obese subjects).

### Treatment protocol and follow-up

All recruited patients underwent standard clinical-laboratory and US examinations on the same day within 1 week before biologic therapy introduction (baseline). Thirty-five patients starting treatment with a TNF inhibitor on stable csDMARD background for ≥3 months (adalimumab, *n* = 25; etanercept, *n* = 7; certolizumab pegol, *n* = 2; golimumab, *n* = 1) were considered for a prospective proof-of-concept analysis with complete examinations at weeks 4 and 24. Follow-up monitoring and treatment decisions were based on standard of care, without knowledge of study findings. By the end of follow-up, four patients discontinued the biological DMARD due to adverse events (*n* = 3) or surgery (*n* = 1). At week 24, patients were categorized as good vs moderate/nonresponders according to the DAS28 and the European League Against Rheumatism (EULAR) response criteria [27]. Patients switching to a different biologic due to primary failure (*n* = 1) before week 24 were considered nonresponders.

### Joint PDUS

Joint PDUS was performed by a single experienced rheumatologist ultrasonographer, unaware of clinical and LN PDUS data, using a GE Logiq 9 scanner (General Electrics Medical Systems, Milwaukee, WI, USA) with a multifrequency linear array transducer (8–15 MHz), according to the EULAR guidelines [28]. A 12-joint assessment model, including transverse and longitudinal scanning of the dorsal view of bilateral wrists (radiocarpal, ulnocarpal, radioulnar, and midcarpal joints) and metacarpophalangeal joints (I–V), was applied [29, 30]. Power Doppler (PD) settings were calibrated to maximise sensitivity as described previously [30] and taken as constant for the same joint in different patients.

Gray-scale (GS) (synovial hypertrophy and/or synovial fluid according to the OMERACT definitions [31]) and synovial PD were graded in each joint through independent semiquantitative (0–3) scales [29, 32]. Two cumulative indices (12-joint GS index and 12-joint PD index) were then calculated at each US assessment as the bilateral sum of either GS or PD grades obtained from each joint (range 0–36) [29, 32].

### Axillary LN PDUS: methods and settings

LN PDUS was performed with the same scanner and transducer adopted for joint US by a single radiologist with >5 years’ experience in breast-axillary sonography, having no access to subject category (RA control), clinical, and joint PDUS data. US examination started, after 5 minutes of rest in a supine position, from the lower part of the axilla and continued upward toward the axillary fossa (pectoral, central, subscapular, and lateral regions) through a maximum scanning time of 5 minutes per side. PD sonography was performed using standardized settings calibrated for high sensitivity with a low wall filter to allow detection of vessels with low blood flow. The pulse repetition frequency was 800 Hz and medium persistence was used. Color gain was set just below the level at which noise artifacts appeared [33].

### Axillary LN PDUS: parameters and grading

Each LN was studied with two-plane scanning. B-mode images (with electronic measurements) and videos of the dynamic PD assessment were then recorded for the analysis of LN volume, structure of the lymphocyte-rich cortex and local perfusion.

**Fig. 1:**
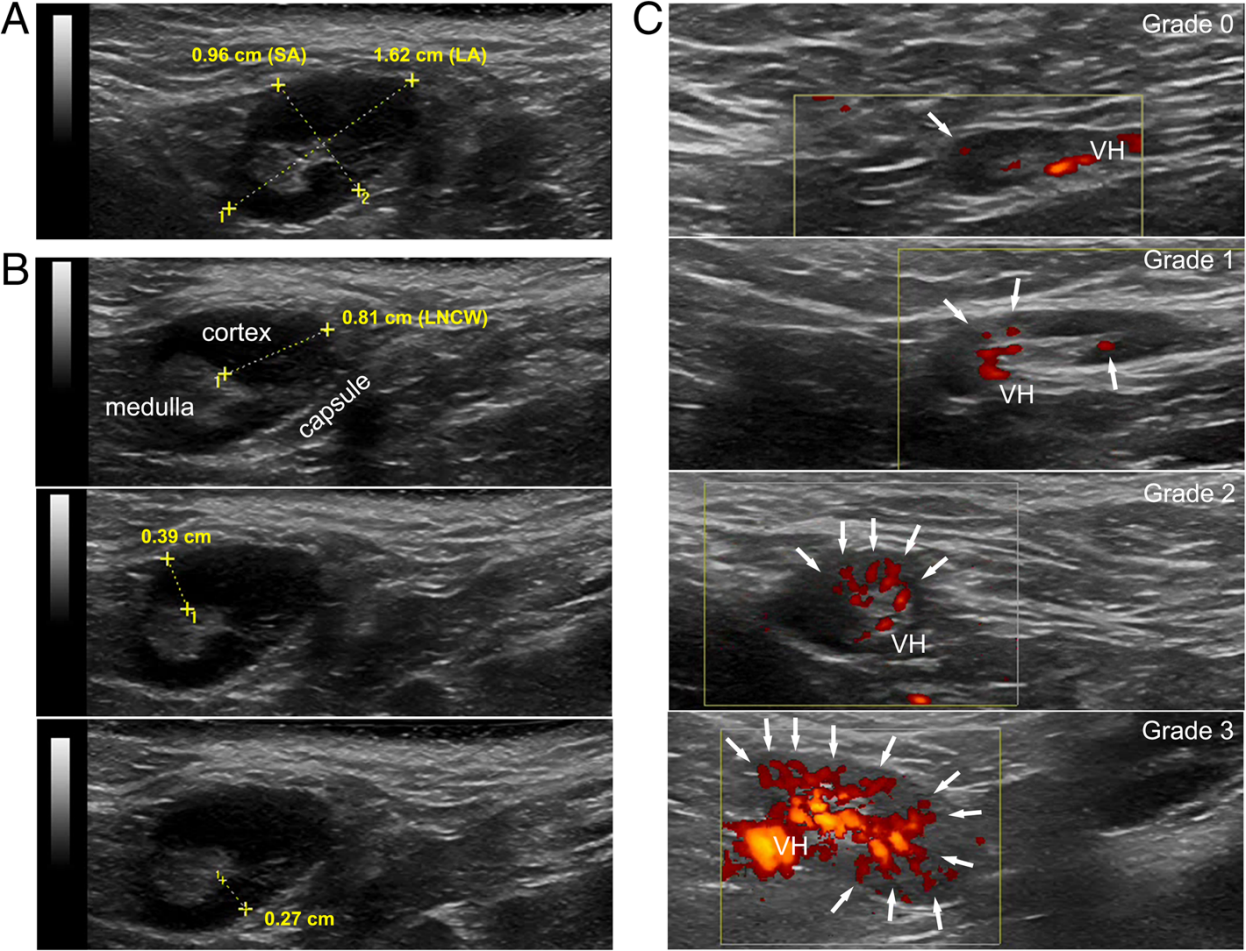
B-mode and power Doppler parameters assessed in axillary LNs by ultrasonography. **a** Representative image of an axillary LN showing the short axis (*SA*) and long axis (*LA*) used for volume calculation (see Methods for description of the formula). **b** Representative images of the same axillary LN showing three independent cortical measurements parallel to LN axes (upper, middle, and lower panels). The major of the cortical measurements (presented in the upper panel) is defined as lymph node cortical width (*LNCW*). The capsule, the hypoechoic cortical area (cortex), and the hyperechoic medullary region (medulla) of the LN are outlined. **c** Representative images of the PD grading system. Four different axillary LNs characterized by progressive PD grades with progressive involvement of the cortical region are shown. *White arrows* indicate the number of PD-positive signals in the cortex. *VH* vascular hilum (anatomic entry site of blood vessels into the node)

Lymph node volume (LNV) was estimated by the ellipsoid formula 4/3π *a* ^2^ *b*, where *b* is the radius on the greatest detected dimension (LN long axis (LA)) and *a* is the radius on its largest orthogonal axis (LN short axis (SA)) [34] (Fig. 1a). Lymph node cortical width (LNCW) was defined as the maximum cortical measurement (from the medulla–cortex interface to the capsule) parallel to the LN axes [35] (Fig. 1b). LNV and LNCW were measured as continuous variables and converted into robust (0–3) semiquantitative scores set on the upper limit of normal (ULN, mean value + 2SD of controls [36]) as the reference threshold: LNV grade 0 = normal (≤0.65 cm^3^, ULN), grade 1 = mild LN hypertrophy (>1 ≤ 2 ULN), grade 2 = moderate (>2 ≤ 3 ULN), and grade 3 = high (>3 ULN); and LNCW grade 0 = normal (≤4 mm, ULN), grade 1 = mild cortical expansion (>4 ≤ 5 mm), grade 2 = moderate (>5 ≤ 6 mm), and grade 3 = high (>6 mm).

Vascular perfusion was graded directly on a semiquantitative scale [37] based on the progressive degree of PD signal [38] detectable within the LN cortex (central and peripheral LN regions according to Steinkamp et al. [37]): grade 0 = absent/minimal cortical flow (reference for calibration: 0–1 PD+ cortical signals), Grade 1 = mild (2–3), grade 2 = moderate (4–5), and grade 3 = high (≥6) (Fig. 1c). Videos of the dynamic assessment are available in Additional files 1, 2, 3, and 4. PD grades were assigned independently (through consensus for discrepancies) by two trained radiologists blind to subject category, clinical-joint US data, and chronological order of the records.


Additional file 1: Assessment in PD mode of an axillary LN graded 0 for perfusion. A static frame of this video is shown in Fig. 1c (upper panel) (WMV 7010 kb)
Additional file 2: Assessment in PD mode of an axillary LN graded 1 for perfusion. A static frame of this video is shown in Fig. 1c (mid-upper panel) (WMV 2554 kb)
Additional file 3: Assessment in PD mode of an axillary LN graded 2 for perfusion. A static frame of this video is shown in Fig. 1c (mid-lower panel) (WMV 2350 kb)
Additional file 4: Assessment in PD mode of an axillary LN graded 3 for perfusion. A static frame of this video is shown in Fig. 1c (lower panel) (WMV 5432 kb)


For each individual, three bilateral cumulative indices (LNV index, LNCW index, LNPD index) were then calculated as the sum of the maximum grade of either LNV, LNCW, or LN PD detected in the right and left axilla (range 0–6). Patients without detectable LN were assigned score 0.

### LN PD grading reliability and digital image analysis

Within-scan inter-reader reliability of the LN PD grading was preliminary evaluated after two calibration sessions on external cases, by comparing the independent scores of the two raters on a set of 40 videos randomly selected by a study investigator from baseline examinations [39]. Within-scan intra-reader reliability was assessed by blinded rescoring of the same videos in a different order 3 months later.

Quantitative analysis of the LN PD signal was performed by digital image analysis (DIA) [33, 37]. Three snapshots from each video were captured at 5-second intervals by an experienced operator unaware of the semiquantitative grade. The mean percentage of color pixels (color fraction (CF)) relative to the pixels of the total LN area (selected manually as region of interest (ROI)) was calculated by ImageJ (NIH, MD, USA), and defined as the PD relative signal.

### Statistical analyses

Demographic and clinical data were presented with mean and SD, median and interquartile range (IQR), or relative frequencies, as appropriate. Reliability of LN PD grading was calculated by exact agreement and weighted kappa statistics. Differences between groups were compared by Mann–Whitney test and chi-square statistics. Correlations between LN US and patients’ variables were computed by Spearman’s rho correlation coefficients. Treatment effects on joint/LN US characteristics at 4 and 24 weeks were investigated by Friedman test with multiple-comparison post-hoc testing. Longitudinal relationships between changes in LN scores and joint US parameters or DAS28 (external responsiveness) was assessed by linear regression [40]. Predictive analyses for response to therapy were performed by logistic regression adjusted for possible confounders. All statistics were based on MedCalc® version 12.7.0.0, and the level of significance was set at 0.05.

## Results

### Reliability of nodal perfusion semiquantitative assessment

To evaluate the applicability of the semiquantitative grading system devised for LN PD measurement, its precision and relationship with digital analysis of LN vascularity was preliminarily scrutinized in a selection of videos.

Reliability exercises showed good strength of agreement with weighted kappa values of 0.77–0.84 and exact agreement of 75–80 % for inter-rater and intra-rater assessments respectively. Objective analysis of PD relative signal (% of total area of the node covered by PD+ vessels) quantitatively measured by DIA on static frames confirmed a linear relationship with raters’ discrimination of grades (*p* < 0.001; Kruskal–Wallis test). More details of grading reliability and digital image analysis are presented in the graphs available in Additional file 5.

### LN sonographic changes are restricted to a subset of patients with active RA

Palpable axillary LNs were identified in 9/40 RA patients (22.5 %; median LN number (range): 0 (0–3)) and in 3/20 controls (15 %; 0 (0–1)). Blind US assessments performed on the same day proved highly increased sensitivity, allowing LN measurement in 36/40 patients (90 %) and 17/20 controls (85 %), without significant differences in terms of LN number between groups (3 (0–9) vs 2 (8–0); *p* = 0.327; Mann–Whitney test). Clear-cut heterogeneity was instead captured through the application of the semiquantitative gradings. Increased perfusion, global hypertrophy, and cortical enlargement compared with controls were indeed recognized in a variable number of axillary stations in patients with RA, and determined significant sonographic differences, at population level, between patients and controls (LNV index, median (range): 0 (0–6) vs 0 (0–0), *p* = 0.009; LNCW index: 0 (0–6] vs 0 (0–0), *p* = 0.009; LNPD index: 1 (0–6) vs 0 (0–2), *p* = 0.039; Mann–Whitney test). Similar results were obtained when morphological parameters were analyzed as continuous variables taking into account the LN with maximum value in each subject: LNV, median (range): 0.43 cm^3^ (0.1–5.1) vs 0.18 cm^3^ (0.1–0.6), *p* = 0.004; LNCW: 3 mm (1.3–10.2) vs 2.6 mm (1.3–3.7), *p* = 0.053 (Mann–Whitney test).

**Fig. 2:**
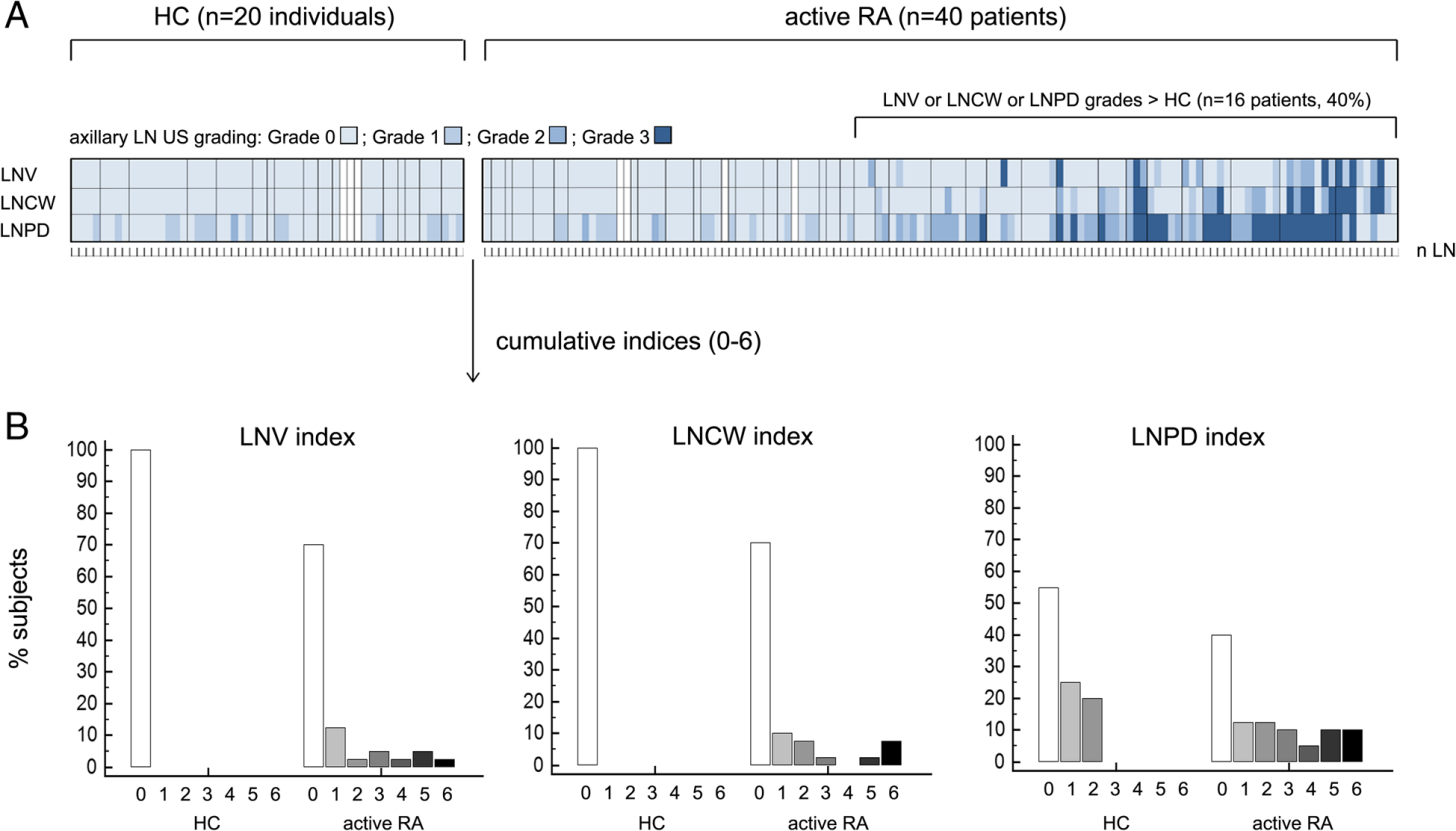
Ultrasonographic characteristics of axillary LNs in rheumatoid arthritis (*RA*) patients at baseline and in controls. **a** Map of ultrasonographic characteristics of all examined LNs. Each individual LN (indicated under the map, *n LN*) is graded 0–3 for lymph node volume (*LNV*), lymph node cortical width (*LNCW*), and lymph node power Doppler (*LNPD*) (see Methods for description of grading system). Individual LNs of each single subject are grouped together using map separators (*vertical black lines* inside the map separate different subjects). *White vertical bars* indicate subjects in whom no measurable LNs were found. RA patients with LNs displaying LNV, LNCW, and LNPD grades exceeding the threshold of healthy controls (*HC*) are clustered together on the right side of the map. Note the restriction of LN alterations in a patient subgroup. **b** Distribution of HC and RA patients according to the LNV, LNCW, and LNPD cumulative indices (sum of maximum detected grade for each parameter in the right and left axilla, see Methods for description). Note the differences between RA patients and HC and the degree of variability captured by each index (presented in separate graphs)

Within the RA group, nodal alterations were not uniformly distributed. Rather, they clustered within a tangible patient subgroup in which multiple nodes were frequently involved (Fig. 2a). The degree of variability captured by each cumulative index is shown in Fig. 2b.

### LN sonographic characteristics are partially influenced by active synovitis in ipsilateral joints

Neither the number of detected LNs nor any of the LN indices was related to IgG anti-citrullinated protein antibodies or IgM rheumatoid factor titers in the whole group or in seropositive cases (Table 2), and no significant differences were present between patients stratified according to autoantibody positivity (data not shown). No relationship was observed between LN parameters and the DAS28, acute phase reactants, patient’s reported clinical/functional outcome measures (data not shown), and objective or semiobjective clinical assessment of joint involvement (Table 2).

**Table 2:** Correlations between lymph node indices and patient characteristics at baseline

	Spearman’s rho correlation coefficient (95 % CI)		
	LNV index	LNCW index	LNPD index
Autoimmune status			
IgG ACPA (U/ml)^a^	−0.07 (−0.38 to 0.25) *p* = 0.677	−0.16 (−0.45 to 0.16) *p* = 0.321	−0.04 (−0.35 to 0.27) *p* = 0.790
(ACPA-positive patients)	0.04 (−0.41 to 0.47) *p* = 0.874	−0.20 (−0.59 to 0.27) *p* = 0.402	−0.12 (−0.54 to 0.34) *p* = 0.605
IgM RF (U/ml)	−0.01 (−0.33 to 0.30) *p* = 0.936	0.05 (−0.27 to 0.36) *p* = 0.752	0.09 (−0.23 to 0.40) *p* = 0.568
(RF-positive patients)	0.08 (−0.40 to 0.52) *p* = 0.760	0.20 (−0.29 to 0.61) *p* = 0.414	0.23 (−0.27 to 0.63) *p* = 0.361
Joint assessment—clinical			
TJC28	0.01 (−0.30 to 0.33) *p* = 0.945	0.01 (−0.30 to 0.33) *p* = 0.929	−0.02 (−0.33 to 0.30) *p* = 0.910
TJC (ipsilateral)^b, c^	−0.02 (−0.34 to 0.29) *p* = 0.876	0.00 (−0.32 to 0.31) *p* = 0.994	−0.07 (−0.38 to 0.25) *p* = 0.656
SJC28	0.14 (−0.18 to 0.44) *p* = 0.394	0.24 (−0.08 to 0.52) *p* = 0.142	0.24 (−0.08 to 0.51) *p* = 0.147
SJC (ipsilateral)^b, c^	0.15 (−0.17 to 0.44) *p* = 0.366	0.25 (−0.07 to 0.53) *p* = 0.121	0.21 (−0.11 to 0.50) *p* = 0.189
Joint assessment—PDUS			
12-joint GS index	0.30 (−0.02 to 0.56) *p* = 0.063	0.33 (0.02–0.59) ***p*** **= 0.039**	0.36 (0.05–0.60) ***p*** **= 0.026**
GS index (ipsilateral)^b^	0.15 (−0.17 to 0.45) *p* = 0.347	0.39 (0.08–0.62) ***p*** **= 0.015**	0.25 (−0.06 to 0.53) *p* = 0.117
GS index (contralateral)	0.05 (−0.27 to 0.36) *p* = 0.767	0.21 (−0.11 to 0.49) *p* = 0.204	0.14 (−0.18 to 0.44) *p* = 0.392
12-joint PD index	0.46 (0.17–0.68) ***p*** **= 0.003**	0.31 (0.00–0.57) *p* = 0.053	0.35 (0.04–0.60) ***p*** **= 0.029**
PD index (ipsilateral)^b^	0.40 (0.10–0.64) ***p*** **= 0.011**	0.42 (0.12–0.65) ***p*** **= 0.007**	0.36 (0.05–0.61) ***p*** **= 0.024**
PD index (contralateral)	0.16 (−0.17 to 0.45) *p* = 0.340	0.16 (−0.17 to 0.45) *p* = 0.342	0.19 (−0.13 to 0.48) *p* = 0.235

*p* <0.05 values are highlighted bold

^a^ACPA titers >340 U/ml were not further diluted

^b^Correlations between LN and joint parameters in nondominant arm

^c^Joint count restricted to I–V metacarpophalangeal joints, I–IV proximal and thumb interphalangeal joints, wrist, elbow, shoulder

*CI* confidence interval, *LNV* lymph node volume, *LNCW* lymph node cortical width, *LNPD* lymph node power Doppler, *ACPA* anti-citrullinated peptide antibodies, *RF* rheumatoid factor, *TJC28* tender joint count in 28 joints, *SJC28* swollen joint count in 28 joints, *PDUS* power Doppler ultrasonography, *GS* gray scale, *PD* power Doppler

On the contrary, when sensitive PDUS imaging of the synovium was applied, significant correlations were consistently detected both for GS and PD indices (Table 2). Correlations between synovial PD and LN scores were confirmed and strengthened over ipsilateral compartments, but lost across contralateral sides (Table 2), pointing to active regional joint pathology as a trigger for the observed sonographic changes in axillary nodes.

Despite this general agreement, however, PDUS imaging of the joints and axillary LNs did not appear to provide overlapping information. Evidence for LN parameters exceeding the threshold of controls was restricted to 16/40 patients (40 %) vs 25/40 cases (62.5 %) in which active (PD+) synovitis was detected (*p* = 0.073; chi-squared test). Even within patients characterized by moderate to high joint PD scores (≥4; median value of the PD score among PD+ subjects), a sizable proportion of the cases (6/14, 42.9 %) displayed LN parameters strictly below the normality cutoff value, suggesting that active synovitis and LN remodeling were cross-sectionally captured as correlating but not redundant processes.

### LN alterations are responsive to TNF blockade

To challenge these data from a dynamic perspective, plasticity of baseline LN status was examined across 24 weeks, addressing its relationship with synovitis changes and disease activity variations upon anti-TNF treatment.

**Fig. 3:**
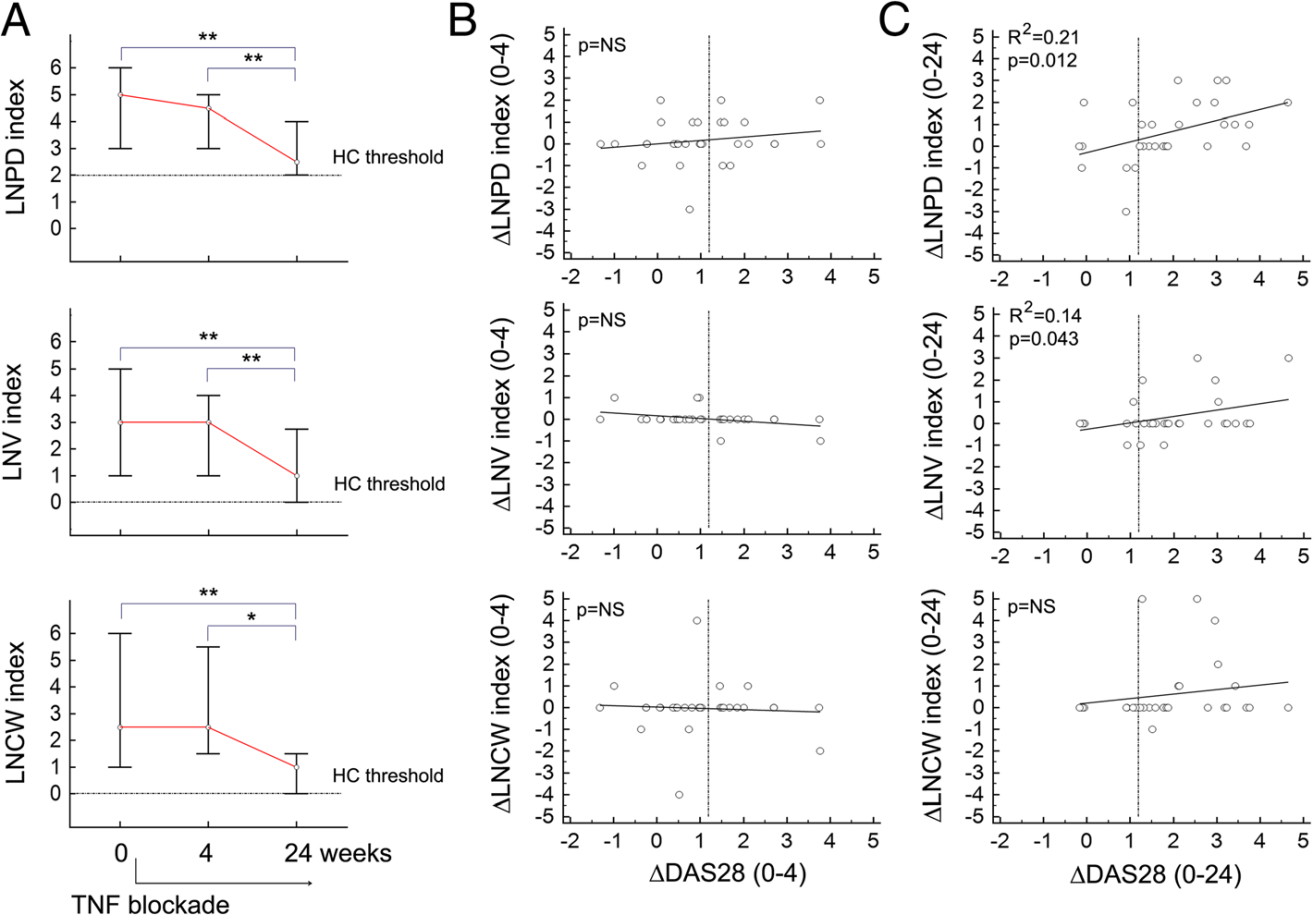
Responsiveness of axillary LN ultrasonographic characteristics. **a** Variation of the subject-related cumulative indices for lymph node power Doppler (*LNPD* index), volume (LNV index), and cortical width (LNCW index) in patients with RA at earlier (4 weeks) and later (24 weeks) time points after institution of treatment with anti-TNF. Median values (*circles*) and 25th–75th percentiles (*error bars*) are presented for each time point. In patients with baseline LN indices above the threshold of healthy controls (*HC*) (*horizontal dotted lines*) significant reductions were observed after 24 weeks but not 4 weeks. *p <0.05, **p <0.01; Friedman test and post-hoc analysis for pairwise comparisons (LNPD index, *n* = 10; LNV index, *n* = 7; LNCW index, *n* = 8). **b, c** Scatter diagrams and regression lines showing the relationship between variations (Δ) from baseline of the LN indices (LNPD, LNV, LNCW) and the 28-joint Disease Activity Score (*DAS28*) at 4 weeks **b** and 24 weeks **c**. Each dot represents one patient (*n* = 30 with complete assessments at baseline, week 4, and week 24). Positive values on the axes of the graphs indicate reduction of the parameters (positive Δ). R^*2*^ linear regression’s coefficient of determination, *LNCW* lymph node cortical width, *LNV* lymph node volume, *TNF* tumor necrosis factor

TNF inhibition induced a prompt response at joint level, with early and stable effects on synovial PD+ alterations (12-joint PD index, median (IQR): baseline, 5 (2–11); week 4, 1.5 (0–8), *p* < 0.01 vs baseline; week 24, 3 (0–4), *p* < 0.01 vs baseline; Friedman test and post-hoc analysis for pairwise comparisons, *n* = 18 with 12-joint PD index > 0 at study entry). Parallel assessment of the axillary LNs revealed average stability of the sonographic pattern in the short term, but could prove its sensitivity to change, showing reduction at 24 weeks of the vascular, volumetric, and cortical scores in patients displaying abnormal parameters at baseline (Fig. 3a). No significant LN modifications (average score upregulation), at any time point, were instead induced by anti-TNF in patients with pretreatment LN indices within the range of controls (data not shown).

External responsiveness, tested against the joint PD index in the whole cohort, confirmed a positive relationship between axillary LN and ipsilateral joint change scores at 24 weeks (∆LNPD: *R* ^2^ = 0.15, *p* = 0.035; ∆LNV: *R* ^2^ = 0.32, *p* = 0.001; ∆LNCW: *R* ^2^ = 0.30, *p* = 0.002; linear regression), but not at 4 weeks (data not shown). Similar, although less systematic, results were obtained assessing changes in DAS28 (Fig. 3b, c).

### Low baseline LN scores are negatively associated with clinical response to TNF inhibitors

**Fig. 4:**
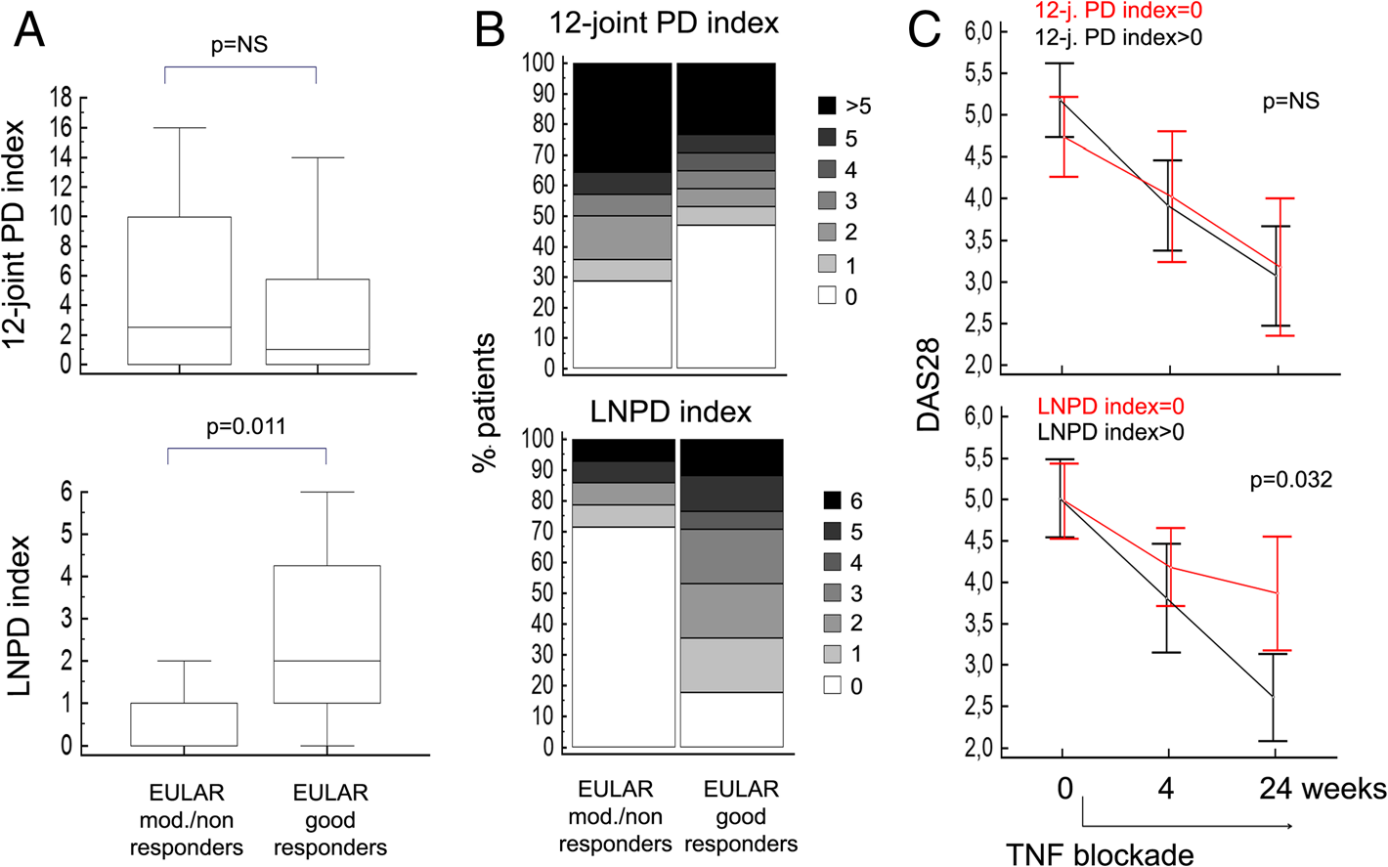
Baseline joint and axillary LN ultrasonographic characteristics in relationship to clinical outcome. **a** Box-and-whisker plots showing baseline differences in the 12-joint power Doppler (*PD*) index (*upper panel*) and the lymph node (*LN*) PD index (*bottom panel*) in patients with RA stratified according to the European League Against Rheumatisms (*EULAR*) response criteria at 24 weeks following institution of anti-TNF treatment. Mann–Whitney test (*n* = 31). **b** Bar charts showing the prevalence of different scores at baseline for the 12-joint PD index (*upper panel*) and LNPD index (*bottom panel*). Each score is presented with a different gray-scale tonality. Enrichment of LNPD index score = 0 (*white*) in patients failing to achieve EULAR good response. **c** Mean (95 % confidence interval) of the 28-joint Disease Activity Score (*DAS28*) during follow-up in patients stratified according to the presence/absence of PD activity in the joints (*upper panel*) and LN (*bottom panel*) at baseline. Different clinical dynamics across 24 weeks of patients exhibiting LNPD index score = 0. Repeated-measures ANOVA in patients with complete assessments at baseline, week 4, and week 24 (LNPD index = 0, *n* = 12; LNPD index > 0, *n* = 18). *TNF* tumor necrosis factor

At 24 weeks, 17 out of 31 patients (54.8 %) achieved a good EULAR response (11/17 reaching remission according to the DAS28) [27], whilst 14 (45.2 %) were moderate/nonresponders. As expected, retrospective evaluation of patients with different treatment outcomes failed to reveal any significant difference in baseline DAS28, patient’s assessment of disease activity, the 28-tender/swollen joint counts, or US synovitis degree (GS and PD indices) (data not shown and Fig. 4a, b).

Of note, consistent diversity could instead be captured through US imaging of the LNs, as inferred by the sharply lower number of detectable LNs (median (IQR): 2 (1–3) vs 4 (2–5.25), *p* = 0.017; Mann–Whitney test) and the lower perfusion scores observed in the moderate/nonresponder group (Fig. 4a, b). Pretreatment LNPD index = 0 (i.e., no cortical PD signal bilaterally) discriminated prospective RA patients characterized by a significant lower reduction in the DAS28 during follow-up (Fig. 4c), turning out to be a negative predictor of good EULAR response, independent of joint PD grade (odds ratio = 0.04, 95 % confidence interval = 0.01–0.35, *p* = 0.004; logistic regression). This result remained significant even after adjustment for age, sex, disease duration, glucocorticoid comedication, and baseline DAS28. Neither the LNV index nor the LNCW index showed similar associations.

## Discussion

We demonstrate that superficial LNs can undergo PDUS-measurable structural and perfusion changes in course of RA; that these changes reflect the existence of an ongoing interactivity with peripheral inflamed sites; and that quantitative analysis of LN status may provide specific information, not captured by standard assessment of the joint. Collectively, these data offer first-time indication of the rationale of PDUS evaluation of the LN as a complementary platform for assessment of the disease and lend direct support to the role of the JLS as a component of RA inflammatory process.

Hand joints and wrists represent the most valuable site for clinical, US, and radiographic examinations across progressive phases of the disease. Based on this concept, we designed this study focusing on the axillary LNs, an easily traceable lymphoid complex receiving terminal lymphatic drainage from the whole forearm, both directly (through deep lymphatics) and across the epitrochlear stations, through superficial lymphatics of the medial compartment [10, 41].

To delineate the actual spectrum of axillary LN sonographic variability, we developed a semiquantitative grading approach and exploited it to measure the structural and functional status of individual LNs, focusing on parameters subjected physiologically to dynamic changes (volume, cortical morphology, and local perfusion). Comparative analyses between active RA patients and healthy individuals proved the discriminative capacity of the adopted scoring system at population level, and demonstrated the existence of measurable inter-patient heterogeneity for all parameters analyzed.

Cross-sectional detection of differences in patients with long-standing disease could be theoretically related to either active or anamnestic events, including the direct input of ongoing inflammation from peripheral joints, the effect of therapy, or the outcome of a stable pathologic imprinting [23, 42]. Our results based on simultaneous assessment of the LN and synovial PD (a sensitive readout of active inflammation in the joint) [43] could capture the influence of the former through four convergent proof-of concepts: the significant correlation between LN scores and synovial Doppler signal; the specific preservation of these correlations on ipsilateral compartments; the possible reduction of LN alterations upon anti-TNF treatment; and the long-term relationship between LN and synovial PD change scores.

These data thus indicate the possible preservation of dynamic interactivity between the joint and the draining LN in established RA, an ancillary path that may contribute to perpetuation of the immune-pathologic process beyond preclinical and early phases of the disease [44]. US detection of altered structural and perfusion scores in axillary nodes might therefore be a sign of effective lymphatic drainage in the context of an active joint inflammatory process, a model that fits with the possible transfer of joint-derived inflammatory mediators in RA lymph [12] and their role in LN hypertrophy and feed arteriole expansion in vivo [45].

If, on one side, these data provide evidence of the impact of peripheral inflammation on LN challenge, then, on the other, the analysis of single individuals demonstrated that active synovitis in hands and wrists was not necessarily coupled to the expression of US changes in the axillary nodes. Despite moderate-to-high disease activity, LN alterations were indeed clustered within a patient subgroup, more restricted compared with the one in which PD+ synovitis was observed. Of note, this partial discrepancy turned out to be relevant and sharper when disease evolution was analyzed. In particular, despite no predictive information inferable from US or clinical evaluation of the joints [46], extension of the assessment to the LNs allowed capture of differences regarding treatment outcome. Lower LN numbers and perfusion scores at baseline, suggestive of a defective response, were indeed significantly related to poorer disease control.

Recent elegant experiments in the murine system delineate a model that may give a putative explanation of these results. It has indeed been shown that lymphatic drainage in the course of arthritis can be impaired, and that LNs draining inflamed joints can undergo a process of “collapse”. This phenomenon is related to translocation of specific B-cell subsets (Bin, B cells in inflamed nodes) in the paracortical sinuses, and is coupled to decreased PD signal and defective lymphatic flow [47, 48]. Of relevance, LN Bin, whose presence in humans has been proved recently [49], can be removed by systemic B-cell depletion [47], but are marginally affected by anti-TNF [50]. Introduction of treatment in a phase in which a central lymphatic road-block is active might thus limit some of the beneficial effects of anti-TNF that include peripheral lymphangiogenesis [51] and increased lymphatic contraction [50].

We are aware that no conclusions can be drawn on the LN as a biomarker of clinical response to treatment due to the small sample size of this proof-of-concept study. Nevertheless, our cross-sectional and longitudinal analyses consistently demonstrate that PDUS assessment of the joint and the draining LN may provide different perspectives on local pathology. This observation is important, because it defines the rationale of a novel analytical approach to peripheral disease, based on integrated assessment of arthritis and nodal involvement.

Another relevant aspect of this study is the application of a quantitative tool for the sonographic characterization of superficial LNs in an inflammatory context. Because this approach appeared successful for the aims of the current investigation, it is important to emphasize also its possible implementation. In particular, due to the lack of an accessible gold standard for the construct “LN reactivity”, the scores we applied were based on progressive thresholds expressing differences in individual morphological characteristics. The development of composite parameters, based on parallel histopathologic-US analyses and designed on an immunological criterion, is among the potential lines of research that may stem from our observations. Additional studies are also warranted to directly compare US with other imaging approaches in order to define performance/limitations of the technique in the assessment of deep axillary stations and more distal structures, such as epitrochlear LNs.

## Conclusions

In this study, we demonstrate the applicability of PDUS to measure and decipher intrinsic aspects of RA pathology beyond conventional assessment of the joint. The integrated analysis of the joint-draining LN complex may represent a novel approach to better delineate the characteristics and outcomes of peripheral inflammation in patients with RA.

## Additional files

Additional file 5: Figure S1. Showing LN PD grading reliability and digital image analysis. Bar charts showing interrater (**A**) and intrarater (**B**) agreement of the LN power Doppler (*PD*) grading system (*n* = 40 LN videos). **C** Representative images of three serial snapshots of an axillary LN adopted for the assessment of PD relative signal by digital image analysis (see Methods for description). *Yellow perimeter* (region of interest (*ROI*)) and the *red area* (color fraction (*CF*)) represent the whole LN area and the PD-positive area used for pixel calculation. **D** Dot plot showing the calculated PD relative signal and its relationship with the semiquantitative PD grades assigned by raters (independent rating with discrepancies resolved by mutual agreement) (*n* = 40 LN videos). Each *black circle* represents one LN. *Red circles* and *horizontal lines* show the median values of the PD relative signal in each grade. ***p* < 0.01, Kruskal–Wallis test and post-hoc analysis (TIF 2865 kb)

## Abbreviations

Bin: B cells in inflamed nodes
csDMARD: Conventional synthetic disease-modifying anti-rheumatic drug
DAS28: 28-joint Disease Activity Score
DIA: Digital image analysis
EULAR: European League Against Rheumatism
GS: Gray scale
IQR: Interquartile range
JLS: Juxta-articular lymphoid system
LN: Lymph node
LNCW: Lymph node cortical width
LNPD: Lymph node power Doppler
LNV: Lymph node volume
PD: Power Doppler
PDUS: Power Doppler ultrasonography
RA: Rheumatoid arthritis
SD: Standard deviation
TNF: Tumor necrosis factor
ULN: Upper limit of normal
US: Ultrasonographic
