# Supplementary figures and images for: Power Doppler ultrasonographic assessment of the joint-draining lymph node complex in rheumatoid arthritis: a prospective, proof-of-concept study on treatment with tumor necrosis factor inhibitors
Source: Arthritis Res Ther. 2016 Oct 22;18:242. doi: 10.1186/s13075-016-1142-7 (PMC5075165; doi:10.1186/s13075-016-1142-7)

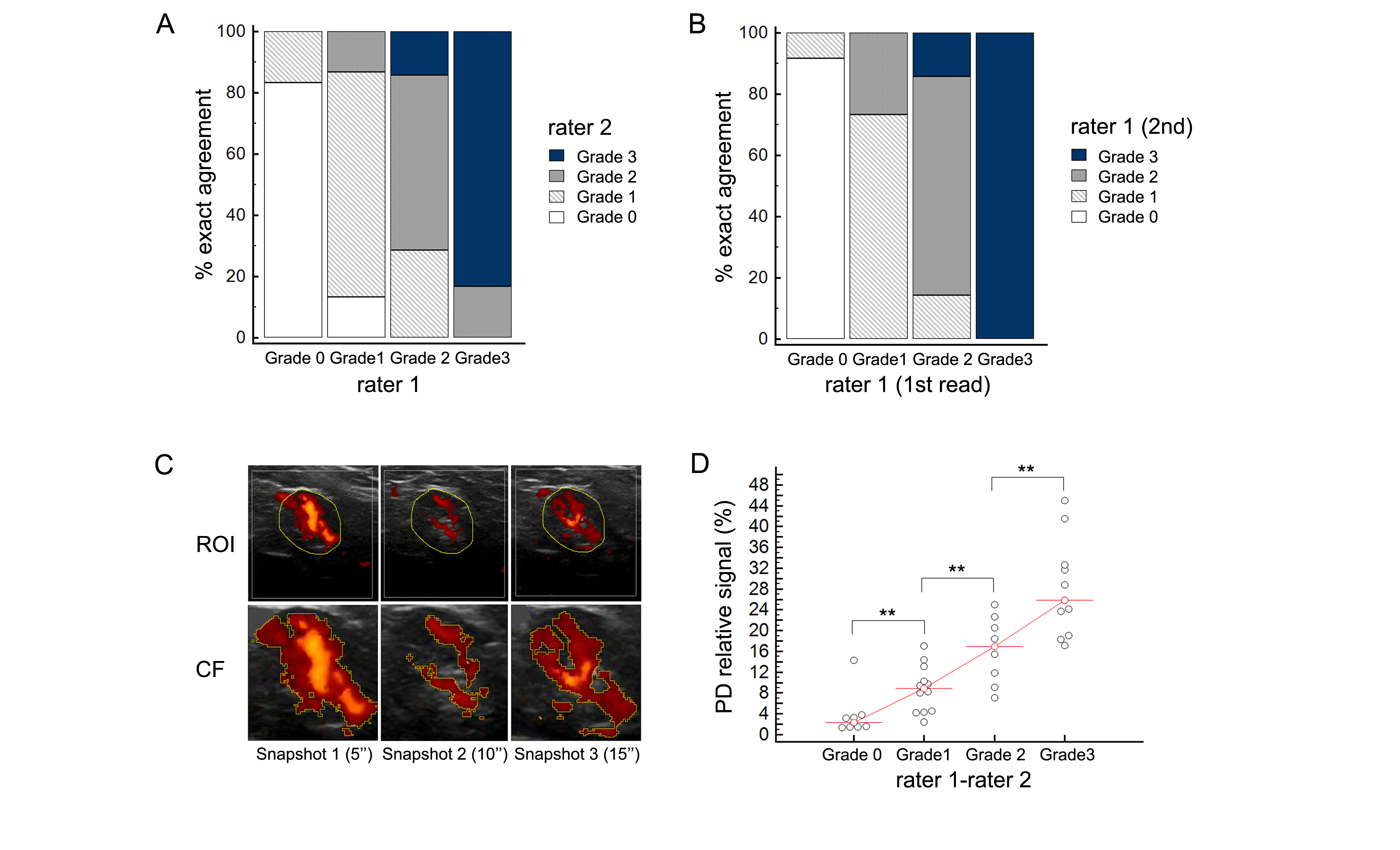

Supplement: Additional file 5: Figure S1. — Showing LN PD grading reliability and digital image analysis. Bar charts showing interrater (A) and intrarater (B) agreement of the LN power Doppler (PD) grading system (n = 40 LN videos). C Representative images of three serial snapshots of an axillary LN adopted for the assessment of PD relative signal by digital image analysis (see Methods for description). Yellow perimeter (region of interest (ROI)) and the red area (color fraction (CF)) represent the whole LN area and the PD-positive area used for pixel calculation. D Dot plot showing the calculated PD relative signal and its relationship with the semiquantitative PD grades assigned by raters (independent rating with discrepancies resolved by mutual agreement) (n = 40 LN videos). Each black circle represents one LN. Red circles and horizontal lines show the median values of the PD relative signal in each grade. **p < 0.01, Kruskal–Wallis test and post-hoc analysis (TIF 2865 kb) [file 13075_2016_1142_MOESM5_ESM.tif]
